# Withdrawal

**DOI:** 10.1002/fsn3.3091

**Published:** 2022-10-19

Withdrawal: “Investigation on *Salmonella enterica*, *Escherichia coli*, and coliforms in beef from Ethiopian abattoirs: A potential risk of meat safety,” by Andarge Zelalem, Kebede Abegaz, Ameha Kebede, Yitagele Terefe, and Jessie L. Vipham, *Food Science & Nutrition*, 2022, 10, 1714–1724. https://doi.org/10.1002/fsn3.2752

The above article, published online in Volume 10, Issue 6 in Wiley Online Library (https://onlinelibrary.wiley.com/doi/full/10.1002/fsn3.2752), has been withdrawn by agreement between the Journal's Editor‐in‐Chief, Prof. Y. Martin Lo, and Wiley Periodicals, LLC. The withdrawal has been agreed because the article was published in error after having been rejected. The authors bear no fault, and we apologize for the error.

